# Reviewed article: Siqueira PFS, Sousa DBP, Moreira AFG, Marchi BS, Teixeira GC, Martins PF, Alves WA. Deaths from infectious respiratory diseases in the Indigenous population: spatial and time series analysis, Minas Gerais, 2000-2023. Epidemiol Serv Saude. 2026:35;e20250565.

**DOI:** 10.1590/S2237-96222026v35e20250565.b

**Published:** 2026-04-20

**Authors:** Gabriel Pavinati

**Affiliations:** 1 Universidade Estadual de Maringá, Departamento de Enfermagem, Maringá, PR, Brazil Universidade Estadual de Maringá Departamento de Enfermagem Maringá PR Brazil

## Peer review A 

## Questionnaire 

Does the manuscript contain new and significant information to justify publication? Yes 

Does the Abstract (Summary) clearly and accurately describe the content of the article? No 

Is the problem significant and concisely stated? Yes 

Are the methods described comprehensively? No 

Are the interpretations and conclusions justified by the results? Yes 

Is adequate reference made to other work in the field? No 

## Manuscript Structure 

Length of article is: Adequate 

Number of tables is: Adequate 

Number of figures is: Adequate 

Please state any conflict(s) of interest that you have in relation to the review of this paper. None 

Interest: Good 

Quality: Good 

Originality: Good 

Overall: Good 

Recommendation: Major Revision 

## Comments 

O artigo intitulado “Tendência temporal e distribuição espacial da mortalidade por doenças respiratórias infecciosas na população indígena em Minas Gerais: análise de séries temporais, 2000-2023” evidencia 271 óbitos registrados em indígenas residentes no estado. A maioria das mortes ocorreu em idosos (62,7%), pessoas do sexo feminino (51,7%) e com nenhuma escolaridade formal (31,4%). Em 61,3% dos casos, a infecção acometeu o trato respiratório inferior, e 50,6% não tiveram agente etiológico especificado. Observou-se tendência crescente de mortalidade em regiões como o Norte do estado (VPA = 18,6%) e redução em regiões como o Sudeste (VPA = -12,4%). A análise espacial identificou aglomerações significativas de óbitos em municípios como São João das Missões, Belo Horizonte e Bertópolis. 

O estudo contribui para o SUS uma vez que evidencia desigualdades territoriais e temporais na mortalidade por infecções respiratórias entre indígenas, fornecendo subsídios para ações direcionadas de vigilância e prevenção. Além disso, reforça a necessidade de políticas públicas específicas e culturalmente sensíveis, com foco na atenção primária e na melhoria da qualidade dos dados sobre mortalidade dessa população, especialmente após à pandemia da covid-19. 

Abaixo faço algumas recomendações com vistas a qualificação do manuscrito e alguns questionamentos para esclarecimentos metodológicos: 

Sugiro a substituição do termo população indígena por povos originários no título, considerando que esta denominação é mais apropriada do ponto de vista político e antropológico, pois reconhece a ancestralidade, a diversidade cultural, histórica, linguística e a organização sociopolítica desses grupos no território brasileiro. 

No resumo, identifiquei a necessidade de ajustes para garantir maior clareza e coerência na apresentação do estudo. A forma como os valores de significância foram inseridos carece de articulação com a narrativa. Recomenda-se reformular trechos como: “Para a análise de tendência, utilizou-se o modelo de regressão por pontos de inflexão (Joinpoint regression)”, e o mesmo se aplica à menção da estatística de Moran, que deve ser devidamente contextualizada. 

Além disso, os resultados estão descritos de maneira abrupta, com a apresentação direta das características predominantes da população estudada, sem uma introdução que situe o leitor. Recomenda-se o uso de conectores e frases de transição que favoreçam a fluidez textual. No que se refere à análise de tendência, sugere-se uma exposição mais objetiva dos achados, indicando claramente as regiões com tendência crescente ou decrescente de mortalidade. 

A expressão “padrão espacial significativo” deve ser melhor explicitada. É essencial indicar onde ocorreram os agrupamentos espaciais (clusters) com maior densidade de óbitos, oferecendo uma descrição geográfica precisa desses padrões. 

Por fim, a frase “alta mortalidade em idosos, desigualdades temporais e territoriais” apresenta-se truncada. Recomenda-se a reescrita para maior completude e coesão, por exemplo: “Os resultados evidenciaram alta mortalidade entre idosos, com expressivas desigualdades temporais e territoriais na distribuição dos óbitos”. 

Os descritores apresentados são suficientes e refletem adequadamente o conteúdo do estudo. 

Nos aspectos éticos, explicitar qual a resolução nacional fundamenta a dispensa apreciação pelo Conselho de Ética em Pesquisa do estudo em questão. 

Na introdução, recomenda-se a inserção de dados epidemiológicos atualizados que descrevam a incidência e a mortalidade por doenças respiratórias infecciosas entre os povos indígenas, de modo a contextualizar a magnitude do problema nessa população e justificar a relevância do estudo. Ademais, a afirmação de que “a limitada produção sobre o tema reforça a necessidade de evidências que subsidiem ações de saúde mais eficazes e culturalmente adequadas” deve ser respaldada por uma revisão da literatura científica. É importante esclarecer o panorama atual das pesquisas sobre o tema, apontando lacunas do conhecimento, limitações metodológicas de estudos prévios ou a escassez de investigações centradas em contextos socioculturais indígenas. Esse reforço teórico contribuirá para demonstrar a originalidade e a pertinência da proposta. 

No método, utilizar para qualificação do relatório de pesquisa o RECORD (Reporting of studies Conducted using Observational Routinely-collected health Data), que é uma extensão do guideline STROBE, voltada especificamente para o relato de estudos observacionais que utilizam dados de saúde rotineiramente coletados. Isso garantirá maior coesão e transparência à pesquisa. 

Especificar que se trata de um estudo ecológico de agregados temporais e especiais. Ainda no delineamento do estudo, já delimitar qual a unidade de análise foi utilizada na pesquisa. 

Na fonte de dados, informar se foi utilizado o Tabnet ou Tabwin para extração de dados e especificar se já foram qualificados ou ainda existem informações preliminares. 

Dentre os critérios de seleção, incluíram-se aqueles que tiveram como causa de morte “não especificadas”. Nesse caso, os autores podem afirmar que esses casos são referentes às doenças respiratórias infecciosas? 

Na análise de dados, a substituição da ausência de óbitos por valores artificiais (como 0,10) nas séries temporais deve ser tecnicamente justificada, uma vez que essa prática pode introduzir viés nas estimativas e comprometer a validade dos resultados. Embora o software Joinpoint aceite valores zero, o uso de log-transformação (comum nesse tipo de análise) torna o log(0) indefinido, razão pela qual alguns optam por inserir valores próximos de zero apenas nesse contexto específico. No entanto, tal decisão metodológica precisa ser explicitada de forma clara no manuscrito, preferencialmente acompanhada de referência que fundamente a escolha. Recomenda-se, ainda, que os sejam discutidos os possíveis impactos dessa imputação artificial nos resultados e nas interpretações da tendência temporal. 

Ainda, sugere-se que os autores apresentem a justificativa para a agregação dos anos utilizada na análise espacial, explicitando os critérios que fundamentaram a divisão em três estratos temporais (2000-2009, 2010-2019 e 2020-2023). É importante esclarecer se essa cisão baseou-se em aspectos epidemiológicos, operacionais ou na distribuição dos dados ao longo do tempo. Ademais, não está claro se a distribuição espacial foi realizada com base na média de óbitos por município (com objetivo de suavizar variações) ou se foram considerados os números absolutos de óbitos em cada período. Essa informação é essencial, sobretudo para a correta interpretação de padrões espaciais e indicadores derivados, como taxas padronizadas ou medidas de autocorrelação espacial. 

Em relação ao método estatístico, solicita-se que os autores especifiquem o número máximo de pontos de inflexão (joinpoints) testados no modelo, uma vez que essa decisão impacta diretamente a complexidade da regressão segmentada e a sensibilidade na detecção de mudanças na tendência. Ademais, não está claro se os dados inseridos no modelo correspondem a valores absolutos de óbitos, médias anuais ou taxas padronizadas por população - informação essencial para a adequada interpretação dos resultados. Embora o manuscrito mencione que slopes com p-valor superior ao nível de significância podem ser considerados para fins de segmentação, não foi explicitado qual valor-p foi adotado como critério para inferência estatística. Por fim, caso tenham sido calculadas taxas ou coeficientes de mortalidade, é imprescindível indicar qual base populacional foi utilizada como denominador, bem como a fonte dessas estimativas (por exemplo, IBGE ou DATASUS). 

Lendo os resultados, parece-me que os autores utilizaram a frequência absoluta para a análise. Nesse caso, precisam ressaltar algumas questões importantes sobre os resultados apresentados: i. a depender da dinâmica demográfica da população estudada, aumentos ou reduções no número de óbitos podem refletir apenas mudanças no tamanho populacional, e não alterações reais na tendência do risco de morte. Tal prática pode, portanto, induzir a falsas interpretações de crescimento ou declínio da mortalidade. Além disso, ii. o uso de contagens absolutas impede comparações adequadas entre regiões ou subgrupos populacionais com diferentes tamanhos, limitando a validade de inferências espaciais ou estratificadas. 

Sugere-se a análise dos resíduos na estatística bivariada, com vistas a indicar a direção da associação observada. 

Ainda, algumas descrições são excessivamente detalhadas (ex: listagens de códigos com distribuição por sexo), o que poderia ser sintetizado em texto e aprofundado somente nas tabelas. 

A alta proporção de “ignorado” ou “sem informação” em variáveis-chave (como agente etiológico e escolaridade) deveria ser discutida com mais ênfase como uma limitação da qualidade do dado no próprio resultado, e não apenas na discussão. 

O uso da frequência absoluta de óbitos sem ajuste populacional precisa ser melhor problematizado, uma vez que não permite avaliar risco nem controlar o efeito de variações populacionais entre anos e regiões. Além disso, a apresentação de intervalos de confiança com extremos idênticos (ex: VPA = 25,0; IC95% = 25,0-25,0) pode indicar erro de modelagem, arredondamento excessivo ou ausência de variabilidade nos dados, devendo ser esclarecida. 

Sobre a distribuição espacial, a sobreposição de tendências crescentes e decrescentes em regiões com poucos eventos pode levar a interpretações instáveis. Recomendaria uma ponderação sobre a viabilidade de agrupar algumas macrorregiões com baixo número de óbitos. 

Recomenda-se que a discussão seja iniciada com um parágrafo introdutório que apresente uma síntese interpretativa dos principais achados do estudo, favorecendo a contextualização dos leitores quanto à relevância e às implicações dos resultados. Observa-se, ainda, a menção ao “maior risco” de adoecimento e óbito por infecções respiratórias na população indígena, porém essa afirmação carece de aprofundamento conceitual e referencial. Sugere-se a inclusão de estudos transversais ou de coorte que evidenciem, com base empírica, a maior vulnerabilidade de povos originários a essas condições. 

No primeiro parágrafo da discussão, destaca-se que a frase “De acordo com o Relatório situacional do DSEI, doenças do aparelho respiratório configuram-se como aquelas com maior mortalidade (7)” está inserida de forma desconexa, sem uma transição lógica com as ideias anteriores. Recomenda-se sua reestruturação ou realocação para outro ponto do texto. 

De modo geral, a discussão apresenta fragilidade na articulação entre os resultados do estudo e a literatura científica, sendo pouco coesa e com escasso diálogo com pesquisas epidemiológicas ou qualitativas prévias. A incorporação de evidências nacionais e internacionais que abordem determinantes sociais, acesso aos serviços de saúde, vulnerabilidades ambientais e práticas culturais pode enriquecer a análise dos desfechos. 

Por fim, a pandemia de COVID-19, embora mencionada pontualmente, não foi adequadamente explorada como um marco sanitário crítico para os achados desta pesquisa, tampouco apontada como limitação metodológica, a despeito de seu impacto sobre o aumento abrupto de óbitos, sobrecarga dos serviços e possíveis inconsistências na notificação. Sugere-se uma discussão mais aprofundada sobre o papel da pandemia nos resultados, bem como sua consideração explícita entre as limitações do estudo.

